# Rapid evaporative ionization mass spectrometry (intelligent knife) for point-of-care testing in acute aortic dissection surgery

**DOI:** 10.1093/icvts/ivac019

**Published:** 2022-02-01

**Authors:** Hannah A Davies, Eva Caamano-Gutierrez, Joscelyn Sarsby, Ya Hua Chim, Steve Barrett, Omar Nawaytou, Amer Harky, Mark Field, Riaz Akhtar, Jillian Madine

**Affiliations:** 1 Department of Cardiovascular and Metabolic Medicine, Institute of Life Course and Medical Sciences, Faculty of Health and Life Sciences, University of Liverpool, Liverpool, UK; 2 Liverpool Centre for Cardiovascular Science, Liverpool, UK; 3 Computational Biology Facility, Liverpool Shared Research Facilities, Faculty of Health and Life Sciences, University of Liverpool, Liverpool, UK; 4 Department of Biochemistry and Systems Biology, Institute of Systems, Molecular and Integrative Biology, Faculty of Health and Life Sciences, University of Liverpool, Liverpool, UK; 5 Centre for Proteomic Research, Institute of Systems, Molecular and Integrative Biology, Faculty of Health and Life Sciences, University of Liverpool, Liverpool, UK; 6 Department of Mechanical, Materials and Aerospace Engineering, School of Engineering, University of Liverpool, Liverpool, UK; 7 Department of Physics, University of Liverpool, Liverpool, UK; 8 Department of Cardiac Surgery, Liverpool Heart and Chest Hospital, Liverpool, UK

**Keywords:** Acute aortic dissection, Rapid evaporative ionization mass spectrometry, Biomechanics, Biochemistry, Tissue integrity, False lumen

## Abstract

**OBJECTIVES:**

Rapid evaporative ionization mass spectrometry (REIMS) can discriminate aneurysmal from normal aortic tissue. Our objective in this work was to probe the integrity of acute dissection tissue using biomechanical, biochemical and histological techniques and demonstrate that REIMS can be used to discriminate identified differences.

**METHODS:**

Human aortic tissue was obtained from patients undergoing surgery for acute aortic dissection. Biomechanical, biochemical and histological assessment was carried out to probe mechanical properties and elastin, collagen and glycosaminoglycan composition of the tissue. Monopolar electrocautery was applied to samples and surgical aerosol aspirated and analysed by REIMS to produce mass spectral data.

**RESULTS:**

Tissue was obtained from 10 patients giving rise to 26 tissue pieces: 10 false lumen (FL), 10 dissection flap and 6 true lumen samples. Models generated from biomechanical and biochemical data showed that FL tissue was distinct from true lumen and dissection flap tissue. REIMS identified the same pattern being able to classify tissue types with 72.4% accuracy and 69.3% precision. Further analysis of REIMS data for FL tissue suggested patients formed 3 distinct clusters. Histological and biochemical assessment revealed patterns of extracellular matrix degradation within the clusters that are associated with altered tissue integrity identified using biomechanical testing.

**CONCLUSIONS:**

Structural integrity of the FL in acute Type A dissection could dictate future clinical distal disease progression. REIMS can detect differences in tissue integrity, supporting its development as a point-of-care test to guide surgical intraoperative decision-making.

**Figure:**
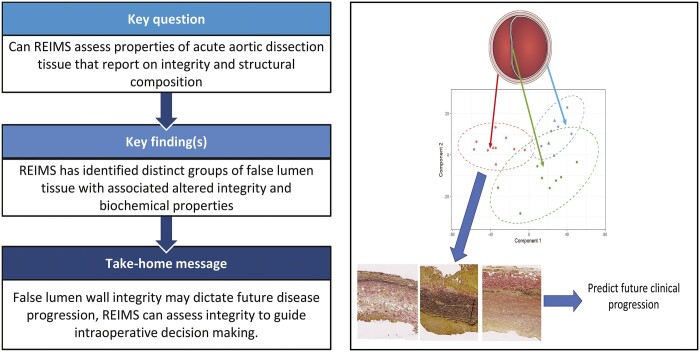

## INTRODUCTION

Acute Type A aortic dissection (AAD) is characterized by blood entering the medial layer of the aortic wall to create a false lumen (FL). This occurs in the root and/or ascending aorta, extending towards the thoraco-abdominal aorta. AAD often results in death at the onset of the pathology and if untreated has an ensuing attrition of 1% per hour over the first 48 h [1]. AAD is treated by surgically replacing the aorta at the origin of the tear with the extent of the surgical repair varying depending upon the clinical presentation, intraoperative findings and patient factors. To date, there has been no method to investigate the integrity of acutely dissected tissues during surgery that might assist the surgeon in decision-making around the extent of surgery. It is often the case that for a proportion of patients whose repair has been restricted to the proximal aorta, there is rapid expansion of the chronically dissected descending thoracic aorta over the first few postoperative years.

Rapid evaporative ionization mass spectrometry (REIMS) is an analytical technique that enables the characterization of human tissues in near real time with no prior sample preparation. REIMS involves analysis of vapour. This can be generated by a hand-held diathermy with hood and suction system known as intelligent knife (iKnife) [2]. iKnife-REIMS (referred to as iKnife) can be used to obtain a molecular profile from tissue *in situ* within seconds. We have previously shown that REIMS can distinguish between control and aneurysmal tissue with, accuracy and precision of 89% and 85%, respectively [3]. Additionally, we showed that aneurysmal tissue from patients with bicuspid and tricuspid valves could be discriminated from normal tissue and each other using REIMS [3].

Here, we investigated the differences in biochemical and biomechanical properties of different areas within *ex vivo* acutely dissected tissue and propose that structural integrity of the FL may dictate future clinical disease progression. We hypothesize that iKnife can identify patterns of type A dissection that may have implications for distal disease progression in the long term, and thus the technology could help to guide surgery.

## PATIENTS AND METHODS

### Ethics statement

This study was ethically approved by Liverpool Bio-Innovation Hub (project approval reference 15-06 and 19-09). The study conformed to the principles outlined in the Declaration of Helsinki and followed STROBE (Strengthening the reporting of observational studies in epidemiology) guidelines. Written informed consent was obtained for all participants.

### Tissue and patient characteristics

**Figure 1:**
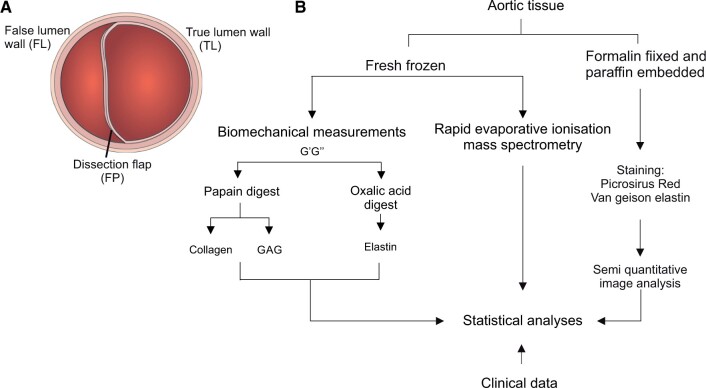
Details of the tissue type and processing used within the study. (**A**) Schematic depicting the 3 types of tissue collected and analysed in the study. (**B**) Workflow detailing the different preparative methods, processes and analyses required for each data set. GAG: glycosaminoglycan

Ascending aortic tissue samples were obtained from patients undergoing emergency aortic repair following DeBakey Type I acute aortic dissection. Where possible, samples from the proximal true lumen (TL) wall representing an adjacent non-dissected section of the aorta composed of all 3 layers, the FL wall comprising the aortic wall remaining following dissection of the inner layer (composed of the adventitia and a portion of media) and the dissection flap (FP) comprising the portion of the aorta that separates (composed of intima and a portion of media) were taken (Fig. 1A).

Patients with known connective tissue disorders were excluded from the study. Tissue was rapidly frozen in super-cooled liquid nitrogen immediately after collection. Patient demographic and clinical characteristics were obtained from the hospital electronic database and recorded.

### Biomechanical measurements

Oscillatory nanoindentation was conducted on the tissue samples to determine localized mechanical properties using a KLA-Tencor Nanoindenter G200 with a DCM-II Head (CA, USA), equipped with a 100 µm flat punch indenter (Synton-MDP Ltd., Nidau, Switzerland). Sixteen indents were applied to the medial layer, indenting the tissue cross-section for 3 pieces of tissue per patient sample. All measurements were performed using a pre-compression of 7 μm, at a frequency of 110 Hz with 500-nm oscillation amplitude. For each indentation, the shear storage modulus (G’) and the shear loss modulus (G˝) were calculated. Full methodological details for this oscillatory indentation method can be found elsewhere [4].

### Biochemical measurements

Aortic tissue was homogenized or digested with papain or oxalic acid as required (see Fig. 1B). For papain digestion, 50 mg of tissue was incubated in 500 μl of papain solution (10 units/ml in 0.1 M sodium acetate, 2.4 mM ethylenediaminetetraacetic acid, 5 mM L-cysteine, pH 5.8) at 60°C overnight. For oxalic acid digestion, 7.5 mg of tissue was incubated in 750 μl of 0.25 M oxalic acid at 100°C for 1 h, and subsequently, the digested supernatant was collected. Hot oxalic acid treatment of residues was repeated another 4 times, using fresh oxalic acid each time, with digests pooled. Collagen was determined by measuring hydroxyproline concentration in the tissue using 1,3-Dimethylbutylamine dye [5, 6]. Glycosaminoglycan (GAG) was measured using dimethyl methylene blue assay [7]. Elastin was measured using Fastin Elastin Kit (Biocolor, Carrickfergus, County Antrim, UK) according to manufacturer’s instructions AQ5. Full methodological details for these assays can be found elsewhere [5].

### Rapid evaporative ionization mass spectrometry

Tissue samples were thawed and a 5 mm punch was removed, wetted using milli Q water and analysed by REIMS (Waters, Wilmslow, Manchester, UK) according to the method previously reported [3]. Using a monopolar diathermy electrosurgical pencil (Erby Medical UK LTD, Morley, Leeds, UK) (iKnife), a power of 35 W was applied at room temperature, the aerosol was aspirated and directed into the REIMS source (Waters, Wilmslow, Manchester, UK). Simultaneously, a lockmass solution of 5 pmol/µl leu-enkephalin in propan-2-ol at a flow rate of 50 µl/min was provided to the REIMS source attached to a Synapt G2-Si (Waters, Wilmslow, Manchester, UK) in resolution mode. Spectra were recorded in negative ion mode from m/z 50–1200 at a scan rate of 1 Hz. Burn events were 10–30 s. Raw data were processed using Progenesis Bridge to convert any uneven burn events into a single pseudo-Gaussian (time, total ion current). Total ion current is the cumulative effect of ions hitting the detector regardless of m/z throughout a single scan event i.e. the sum of all m/z intensities of the spectra at the specific scan. The processed total ion current trace was imported in LiveID and was 35 scans across, with the burn event covering 12 scans reaching a maximum at scan 18.

### Histology and image analysis

FL samples were formalin fixed and paraffin embedded for histological analysis. Six-micrometer sections were stained with Verhoeff–van Gieson (VVG) and Picrosirius Red using standard protocols. Analysis of the relative areas of collagen and elastin in the images was implemented by incorporating a customized VVG stain analysis routine into the general-purpose image analysis software Image SXM [8].

Firstly, the area of tissue within the image was determined. The blue channel of the RGB colour image has the highest contrast between the tissue and the background therefore this is used to create a binary mask of the tissue. The modal pixel value in the image corresponds to (bright) blank slide, by selecting all pixels that have values less than this defines the tissue area. A binary image of the tissue is processed to remove small fragments of tissue lying outside and to close small holes within the main body of tissue. The resultant binary image is used as a mask to define which pixels are part of the tissue and which are not. Only pixels that are within the tissue mask contribute in the subsequent calculations of collagen and elastin.

For the calculation of the area of collagen, an algorithm was implemented to discriminate stained tissue from unstained tissue. Stained collagen can manifest as a range of colours from light pink through shades of magenta (red-blue) to dark red. A figure of merit index was calculated as a weighted sum of the red (R), green (G) and blue (B) pixel values. By selecting judicious weighting factors of:

collagen index=R/50 – G/20+B/30

the resultant index has values −0.75 to 1.0 for non-collagen tissue and 1.0–1.75 for collagen. An image of collagen indices can then be thresholded to create a binary image showing the location of collagen in the tissue sample. Counting the pixels above the threshold gives the area of collagen. The threshold (nominally 1.0) was calibrated for the tissue samples by determining the collagen area from adjacent tissue sections stained with Picrosirius Red and ensuring that the collagen area for the VVG stain gave consistent values.

The calculation of the area of elastin follows the same approach except that the VVG stain results in grey-to-black pixels and so the R, G and B pixel values are equally weighted to generate an elastin index. The index is nominally 0–0.5 for non-elastin and 0.5–1.0 for elastin. The image is thresholded at a value of 0.5. All calculations of percentage area are the ratios of the pixel counts for the collagen or elastin divided by the pixel count in the tissue mask and so spatial calibration of the images is not required. To estimate the errors in the calculations for the areas of collagen and elastin the thresholds were varied either side of the optimal values. Areas that are sensitive (insensitive) to the threshold values result in larger (smaller) errors.

### Statistical analyses

Exploration of the data was carried out using multivariable transformation principle component analysis (PCA) performed on mean centred and scaled data using the prcomp function within the stats package in the statistical software R [9]. Differences between the 3 tissue type groups were further assessed via Dunn Kruskal–Wallis test with *P*-values adjusted with the Holm method using the R package FSA (Fisheries Stock Analysis) [10] for all variables identified as important contributors in the PCA.

Differences between tissue types using REIMS were further explored using partial least squares-discriminant analysis. Partial least squares-discriminant analysis models were created using data split in 80/20 proportions for training/test. This is a standard procedure that refers to the sub-setting of data; 80% of the patient samples were used to fit a model and 20% were used to test the model. The number of components chosen for the model was determined using a 2-fold cross-validation and selecting the number of components that minimize the misclassification error. The test data were used to assess the model and calculate accuracy, sensitivity, recall and f1 for the model (see Ref. [3] for definitions of these terms). Important features of the models were extracted and reported (Variable Importance Projections plots). This process was repeated 50 times generating 50 models and 50 test datasets with different strata of data with average accuracy and sensitivity reported. A second model was created in the same manner by selecting a subset of data based on m/z values with a Variable Importance Projections mean and median larger than 1. Representative score plots and model accuracies are reported, with receiver operating characteristic and area under the curve calculated based on the predicted scores obtained from the refined model on the test data.

To test the hypothesis regarding heterogeneity within the FL group, Ward hierarchical clustering was performed to identify any sub-groups within this cohort. Uncertainty of cluster analysis was assessed calculating *P*-values via multiscale bootstrap resampling using the package pvclust within the R environment [11]. Clusters with an unbiased *P*-value larger than 0.90 were considered as true clusters.

Histological differences identified by image analysis between identified clusters were assessed using Mann–Whitney *U* with adjusted *P*-values presented.

## RESULTS

### Study population and study design

Cohort characteristics and biochemical and biomechanical measurement data are collated in Table 1 and Supplementary Material, Table S1. Ten patients gave rise to 26 acute aortic tissue pieces: 10 FL, 10 FP and 6 TL samples. The median age of the patients was 57 years with a median indexed aortic size of 12.3, 3/10 were female. All 26 pieces were analysed biomechanically and then subsequently biochemically. An additional piece of tissue from all regions/patients was analysed with REIMS.

**Table 1:** Summary patient clinical characteristics collated from electronic patient records

Age [years, median (IQR)]	57 (29)
(Minimum, Maximum)	(27, 75)
Sex (male:female)	7:3
Indexed aorta size [median (IQR)]	12.3 (6.1)
(Minimum, Maximum)	(5.2, 25.5)
Diabetic	0
Hypertensive	2/10
Hypercholesterolaemia	1/10
Family history of aneurysm	3/10

Data are presented as median and interquartile range (IQR) with maximum and minimum values given, or as number of patients.

### Biomechanical and biochemical interrogation of the 3 tissue types

**Figure 2:**
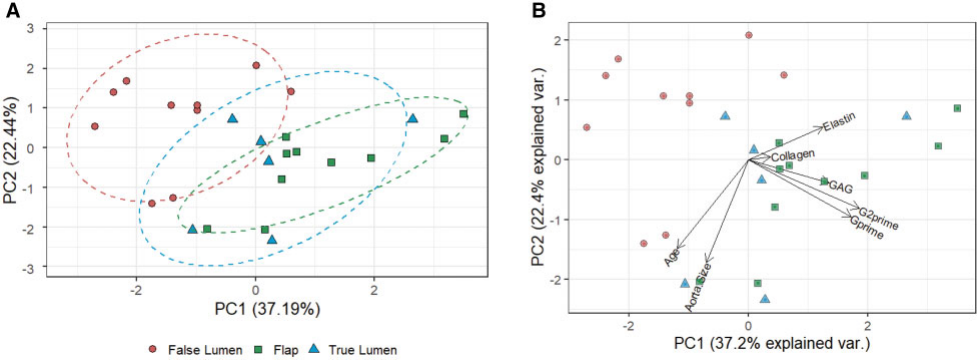
False lumen is distinct compared with the other tissue types. (**A**) Principal component analysis score plot of the biochemical and biomechanical properties of the 3 tissue types, false lumen (red circles), dissection flap (green squares) and true lumen (blue triangles). Ellipses represent 75% of the region around the mean of the points of each group. (**B**) Loadings plot highlighting the analytes and properties that contribute most to the separation of the tissue types.

**Figure 3:**
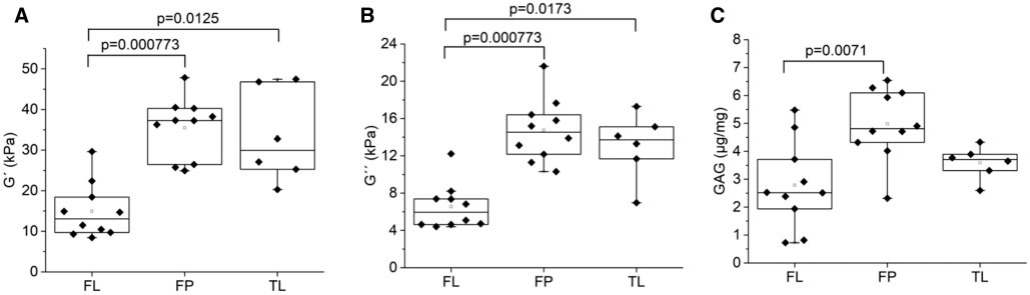
Box plots showing the differences in the key analytes (**A**) G’, (**B**), G˝ and (**C**) GAG, identified in loadings plot separated by tissue type. Data are presented as median values with adjusted *P*-values determined by Dunn Kruskal–Wallis.

In order to explore the differences in the 3 tissue types biomechanical, biochemical and clinical metadata (age and aortic size) were analysed using PCA (Fig. 2A). This unsupervised technique showed that data points of the FL tissues (Fig. 2A, red circles) were largely separated from the FP and TL data points, suggesting that FL tissue was the most distinct from the other 2. The loadings plot for the PCA (Fig. 2B) indicated that the observed separation was largely due to the biomechanical properties G′ (shear storage modulus) and G′′ (shear loss module), and the GAG levels, which were significantly contributing to the separation (Supplementary Material, Table S1). Box plots of these variables showed that G′ and G′′ were significantly lower in the FL group than FP and TL (Fig. 3A and B). This suggests that the FL tissue is more compliant than the other 2 tissue types. GAG pooling in the medial layer of the aorta is thought to contribute to AAD; here, we observed lower levels of GAGs in the FL group when compared with the FP pieces (Fig. 3C).

### Rapid evaporative ionization mass spectrometry

**Figure 4:**
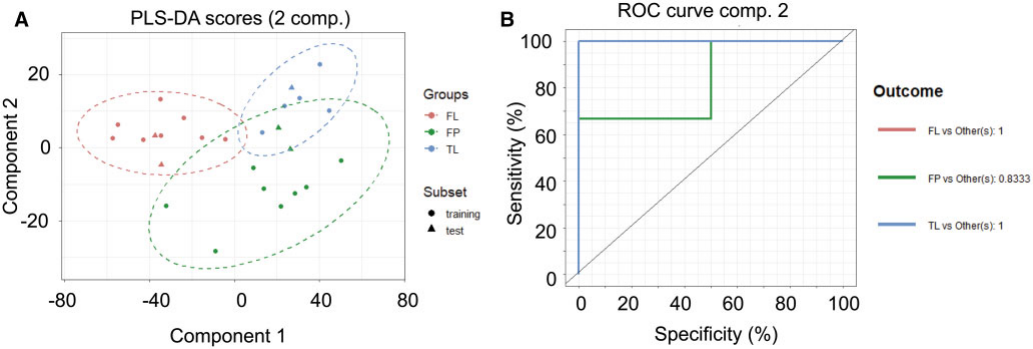
Rapid evaporative ionization mass spectrometry data. (**A**) Score plot of the 2-component model. Points represented for both training (circles) and test (triangles) for all groups; false lumen (red), dissection flap (green) and true lumen (blue) with ellipse representative of the 95% region around the means of each group. The data are consistent with biochemical/biomechanical data analysis indicating false lumen is the most distinct tissue type. (**B**) Receiver operating characteristic curve of the model representing an area under the curve value of 0.963. Fifty models were fitted with average statistics reported in Supplementary Material, Table S2, with area under the curve assessment reported in Supplementary Material, Table S3.

Duplicate *ex vivo* AAD samples were also assessed using REIMS. Firstly, we use REIMS to analyse FL, FP and TL tissues and determine if, like the biomechanical and biochemical analyses it can identify differences in the tissue composition. Figure 4 depicts a representative partial least squares-discriminant analysis of the 3 tissue types analysed with REIMS with the accompanying receiver operating characteristic curve. It shows remarkable similarity with the PCA analysis of the biomechanical and biochemical analysis in Fig. 2A identifying FL tissue as the most distinct. REIMS is able to discriminate FL tissue from the other tissue types with 72.4% accuracy and 69.3% precision (Supplementary Material, Table S2).

### Patterns within the false lumen cohort

**Figure 5:**
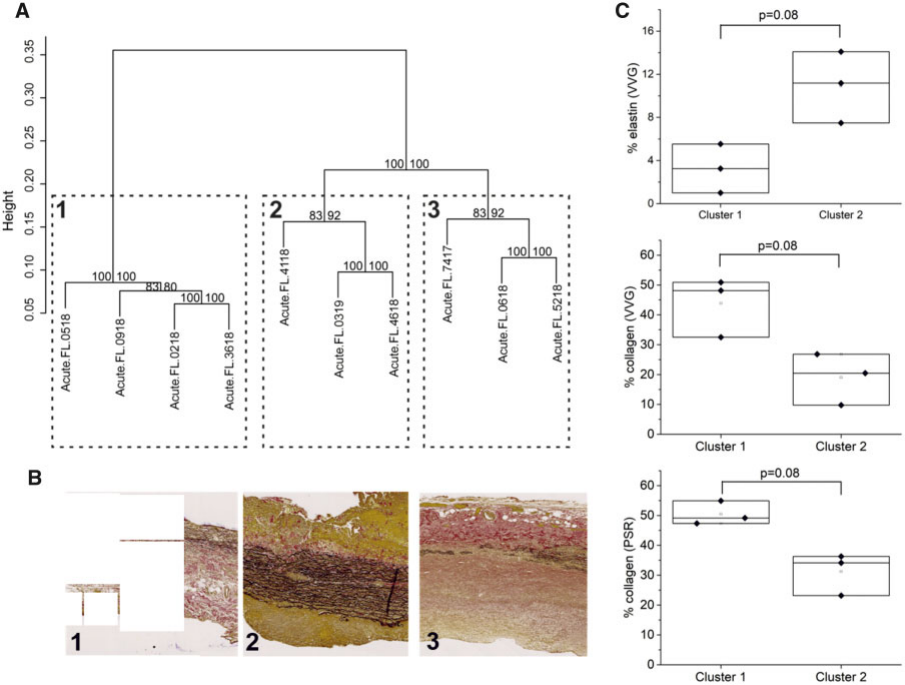
Clustering of false lumen data. (**A**) Ward Hierarchical clustering of REIMS data from the FL group identifies 3 stable clusters. (**B**) Representative VVG stained sections from each of the clusters identified. (**C**) Box plots for percentage of elastin and collagen obtained from image analysis of VVG and PSR stained sections for clusters 1 and 2. Adjusted *P*-values determined by Mann–Whitney *U*. REIMS: rapid evaporative ionization mass spectrometry; VVG: Verhoeff–van Gieson.

Ward hierarchical clustering of REIMS data for FL tissues resulted in 3 stable clusters (Fig. 5A) which pointed towards a highly consistent difference between these 3 groups of tissue. Histological analysis of tissue samples was carried out using VVG images to quantify elastin and collagen as a percentage of total tissue area (Supplementary Material, Table S4). Picrosirius red-stained sections were also analysed for collagen quantification. This histological assessment revealed that samples within cluster 1 had relatively large quantities of collagen deposition with very little elastin. Cluster 2 tissue appeared the most similar to ‘normal’ tissue histologically with relatively high levels of elastin remaining and little collagen (Fig. 5B and C). In contrast, cluster 3 had very little elastin and intermediate levels of collagen present.

## DISCUSSION

The extent of surgical repair of an acute aortic dissection is determined by patient presentation, demographics and presence of intimal tear and aneurysm disease. To date, it has not been possible to objectively measure the integrity of the dissected tissues intraoperatively to aid decision-making. Indeed, dissected tissue is often resected and discarded. Based on our previous investigations of aneurysmal tissue with iKnife-REIMS [3], we hypothesized that acutely dissected tissue may harbour important information that may contribute to intraoperative decision-making. In particular, we felt the FL wall may be key to this as it is often the FL that dilates most in the descending thoracic aorta in the early years following proximal repair.

This work has provided insight into the biomechanical and biochemical characteristics of the different tissues (FL, flap and TL) associated with aortic dissection highlighting that FL is the most distinct with altered biomechanical properties. These biomechanical and biochemical analyses provide important characterization of rare surgical samples that further our scientific understanding of aortic dissection. We have also shown that iKnife-REIMS, an emerging technique that can assess tissue in real time within operating theatres also identifies FL tissue as distinct with 72.4% accuracy and 69.3% precision.

Additionally, REIMS data identify 3 stable clusters within FL tissue that have corresponding patterns of extracellular matrix (ECM) composition. This suggests that REIMS is able to identify ECM differences. Cluster 2 has higher levels of elastin and reduced levels of collagen compared with cluster 1. Collagen-rich regions have been reported to have lower elastic modulus than elastin-rich regions [12]. This trend is consistent with findings on porcine aorta which show that high tissue elastic modulus can be correlated with high elastin density [13]. These studies suggest that elastin is the predominant ECM component for maintaining tissue integrity. Taken together with the data presented here we predict that cluster 2 is likely to be the most stable over time.

Guided by these data, we hypothesize that FL integrity due to alteration of ECM components dictates risk of further aortic pathologies and re-dilation. This suggests that acutely dissected tissue removed at surgery should not be discarded. Instead, it contains useful prognostic information which when analysed by Knife, may guide intraoperative decision-making on the extent of surgery. REIMS analysis is a real-time assessment taking seconds, generating an output instantly. The iKnife may be able to guide surgeons into use of the frozen elephant trunk if it demonstrates that the FL is weak and likely to expand distal to the repair in a short period of time.

### Limitations

Whilst data presented in this study provide insight into the biochemical and biomechanical properties of tissue samples from acute aortic dissection, we acknowledge that sample numbers are low, particularly following cluster analysis. We also highlight that all techniques employed in the study have their own limitations and inherent errors associated with them which require consideration when assessing data for individual variables and not trends across groups. The major limitation of this study, however, is the lack of clinical follow-up data for patients included due to the length of time needed for patients to be re-assessed in clinic which is required to test the hypotheses proposed. We do, however, feel these preliminary data highlight 2 important points to share at an early stage: (i) iKnife could be an important adjunct to surgical decision-making on the extent of surgery for acute Type A aortic dissection repair and (ii) FL tissue characteristics have differing ECM patterns that may contain important prognostic information.

### Future work

This preliminary work will act as a platform for further studies including:

1. Addition of patients to the data set and creation of a library of ‘biosignatures’ which includes sub-groups such as chronic aneurysm, syndromic and non-syndromic disease.
2. Addition of intraoperative *in situ* measurements.
3. Inclusion of the technology in open repair of acute Type B aortic dissection.
4. Follow-up imaging analysis relating patterns of dissection in FL tissue and long-term rates of dilatation in the proximal descending aorta.

### Proposed future clinical application and models of care

We foresee this technology being important in 2 scenarios:

1. Intraoperative decision-making around the extent of surgical resection in repairing an acute Type A aortic dissection.
2. Early follow-up multidisciplinary team discussion of adjunct thoracic endovascular aortic repair in sub-acute phase (2–12 weeks) following acute Type A repair. Based on iKnife analysis and early laboratory analysis of the FL tissue from resected acute Type A proximal aorta, it may be that the team would consider early thoracic endovascular aortic repair in the sub-acute phase.

## CONCLUSION

REIMS data coupled with multivariate analysis can be used to determine differences in patterns of dissection in acutely dissected FL which may have prognostic information. This technology has the potential to transform the care of patients undergoing elective and emergency aortic surgery. Patients undergoing emergency surgery for aortic dissection may have the extent of surgery modified by intraoperative data on the integrity of the FL wall at point of resection and predicted likelihood of further aortic dilation. Or these data may be used to guide multidisciplinary team discussion around adjunctive thoracic endovascular aortic repair in the sub-acute phase following Type A repair.

## SUPPLEMENTARY MATERIAL

Supplementary material is available at *ICVTS* online.

## Supplementary Material

ivac019_Supplementary_Data

## Notes

Presented at the 35th Annual Meeting of the European Association for Cardio-Thoracic Surgery, Barcelona, Spain, 13–16 October 2021.

## Abbreviations

**ABBREVIATIONS**
AAD: Acute aortic dissection
ECM: Extracellular matrix
FL: False lumen
FP: Dissection flap
GAG: Glycosaminoglycan
iKnife: Intelligent knife
PCA: Principle component analysis
REIMS: Rapid evaporative ionization mass spectrometry
TL: True lumen
VVG: Verhoeff–van Gieson
