# Supplementary material for: Rapid evaporative ionization mass spectrometry (intelligent knife) for point-of-care testing in acute aortic dissection surgery
Source: Interact Cardiovasc Thorac Surg. 2022 Feb 1;34(5):833–40. doi: 10.1093/icvts/ivac019 (PMC9070471; doi:10.1093/icvts/ivac019)
Supplement: ivac019_Supplementary_Data [file ivac019_supplementary_data.docx]

**Supplementary Information**

**Supplementary Table 1: Measured parameters displayed as median (interquartile range).** Adjusted p-values determined by Dunn Kruskal–Wallis.

| **Measured parameter** | **False lumen** | **True lumen** | **Flap** | **p-value** |
| --- | --- | --- | --- | --- |
| G' (Kpa) | 13.08 (8.72) | 29.93 (21.56) | 37.27 (13.82) | 0.000556 |
| G'' (Kpa) | 5.95 (2.75) | 13.71 (3.43) | 14.53 (4.24) | 0.000645 |
| GAG (µg/mg) | 2.52 (1.78) | 3.71 (0.58) | 4.81 (1.78) | 0.00873 |
| elastin (µg/mg) | 29.39 (8.16) | 31.78 (19.53) | 34.94 (16.66) | 0.606 |
| Collagen (µg/mg) | 31.30 (15.01) | 31.22 (5.56) | 31.86 (10.29) | 0.809 |

|  | **FP** | | | **FL** | | | **TL** | | |
| --- | --- | --- | --- | --- | --- | --- | --- | --- | --- |
|  | Mean | SD | Median | Mean | SD | Median | Mean | SD | Median |
| Tn | 2.38 | 0.830 | 3 | 1.98 | 0.979 | 2 | 3.48 | 0.814 | 4 |
| Tp | 0.86 | 0.783 | 1 | 1.64 | 0.598 | 2 | 0.34 | 0.479 | 0 |
| Fn | 1.14 | 0.783 | 1 | 0.36 | 0.598 | 0 | 0.66 | 0.479 | 1 |
| Fp | 0.62 | 0.830 | 0 | 1.02 | 0.979 | 1 | 0.52 | 0.814 | 0 |
| Accuracy | 0.648 | 0.183 | 0.6 | 0.724 | 0.185 | 0.8 | 0.764 | 0.184 | 0.8 |
| Precision | 0.645 | 0.335 | 0.667 | 0.693 | 0.256 | 0.667 | 0.463 | 0.456 | 0.333 |
| Recall | 0.43 | 0.391 | 0.5 | 0.82 | 0.299 | 1 | 0.34 | 0.479 | 0 |
| f1 | 0.663 | 0.157 | 0.667 | 0.737 | 0.1582 | 0.667 | 0.847 | 0.223 | 1 |

**Supplementary Table 2: Statistics for model generated using 3786 m/z values.** Mean, standard deviation (SD) and median are given for: True positive (Tp), True negative (Tn), False negative (Fn), False positive (Fp), accuracy, precision, recall and harmonic mean of precision and recall (f1= 2*(precision*recall)/(precision+recall).

**Supplementary Table 3: 95% confidence intervals for area under the curve (AUC).**

| **Model** | **95% AUC, component 2** |
| --- | --- |
| FL/FP/TL, selected variables | FL vs Others (0.971, 1)  FP vs Others (0.971, 1)  TL vs Others (0.957, 1) |

**Supplementary Table 4: Histological data for identified clusters within FL tissue.** Collagen and elastin percentage per tissue area were calculated from VVG and PS (collagen only) stained sections. Data is presented as median and interquartile range (IQR) for samples within each cluster except for cluster 3 where only one sample was available for analysis.

|  | Cluster 1 | Cluster 2 | Cluster 3 |
| --- | --- | --- | --- |
| Collagen (PSR %) | 50.45 (3.96) | 31.18 (7.04) | 46.22 |
| Collagen (VVG %) | 43.84 (9.92) | 19.00 (8.63) | 39.73 |
| Elastin (VVG %) | 3.26 (2.27) | 10.92 (3.32) | 1.19 |
